# Eye Movements and Verbal Report in a Single Case of Visual Neglect

**DOI:** 10.1371/journal.pone.0043743

**Published:** 2012-08-24

**Authors:** Valerie Benson, Magdalena Ietswaart, David Milner

**Affiliations:** 1 Psychology, University of Southampton, Southampton, United Kingdom; 2 Psychology, University of Northumbria, Newcastle, United Kingdom; 3 Psychology, University of Durham, Durham, United Kingdom; Charité University Medicine Berlin, Germany

## Abstract

In this single case study, visuospatial neglect patient P1 demonstrated a dissociation between an intact ability to make appropriate reflexive eye movements to targets in the neglected field with latencies of <400 ms, while failing to report targets presented at such durations in a separate verbal detection task. In contrast, there was a failure to evoke the usually robust Remote Distractor Effect in P1, even though distractors in the neglected field were presented at above threshold durations. Together those data indicate that the tight coupling that is normally shown between attention and eye movements appears to be disrupted for low-level orienting in P1. A comparable disruption was also found for high-level cognitive processing tasks, namely reading and scene scanning. The findings are discussed in relation to sampling, attention and awareness in neglect.

## Introduction

Unilateral neglect is a condition in which patients fail to respond to, orient towards, or report, stimuli located on the side of space contralesional to brain damage in the absence of sensory motor deficits [1]. It is observed most frequently and is of longer duration following damage to the right hemisphere of the brain [2], resulting most commonly therefore, in the left side of space being ‘neglected’. The examination of eye movements should provide insights into attentional and processing impairments in neglect since there is a close relationship between eye movements and cognitive processing (e.g., [3]–[5]) for many tasks.

Although hemi-neglect does not result from an inability to scan the contralesional side of space, early work on eye movements in this area showed deficits in making contralesional saccades (e.g. [6]–[8]). These include multi-stepping hypometric (short) saccades of long latency into the neglected field, failure to fixate upon visual information presented in that field, and failure to report information that may be successfully fixated, in the neglected hemispace. These and later studies have provided evidence for hyperattention to the ipsilesional side in neglect (e.g. [9]–[10]), although hypoattention to information on the left has also been argued to underpin neglect [11]. The effects of contralesional distractors on the time taken to initiate saccades to targets presented in ipsilesional space [8] has resulted in the suggestion that an imbalance in the saccadic system can underpins neglect, and this has received recent support [12].

Exploratory eye movements into the left side of space can be increased following prism adaptation (PA), but without an increase in awareness for stimuli in that space [13] suggesting that the defining perceptual symptoms of neglect are unaffected by low-level visuomotor manipulations. However, awareness for stimuli in the neglected field can increase with PA techniques, but effects are modulated by the categorical nature of the stimuli [14], and are not observed in all cases. Forcing the eyes to scan the neglected field does not guarantee increased awareness for whatever is under inspection. There is also some evidence to suggest that although ‘neglect’ patients may be unaware of information on the left, they may still process such information pre-attentively (e.g. [15]), however this level of processing is insufficient for the information to reach awareness, and hence it goes unreported (cf. the burning house experiment, [16].

To date, evidence from eye movement studies has not conclusively demonstrated whether neglect reflects a failure to look at (sample) relevant information, or a failure to show awareness for (perceptually process) relevant information in the neglected field. Moreover, it remains unclear as to whether any disjoints between sampling and awareness (‘looking’ and ‘seeing’) lie exclusively in the domain of voluntary orienting, or whether the reflexive orienting system is also impaired. This paper reports data from a detailed examination of eye movements in P1, a single case study of hemi-neglect. The aim was to examine awareness (as tapped through verbal responses) and overt behaviour (eye movements) to see whether P1 showed any dissociation between conscious perception for stimuli and saccadic orienting to such stimuli during lower-level oculomotor, and/or higher-level cognitive processing tasks.

## Case History

**Figure 1:**
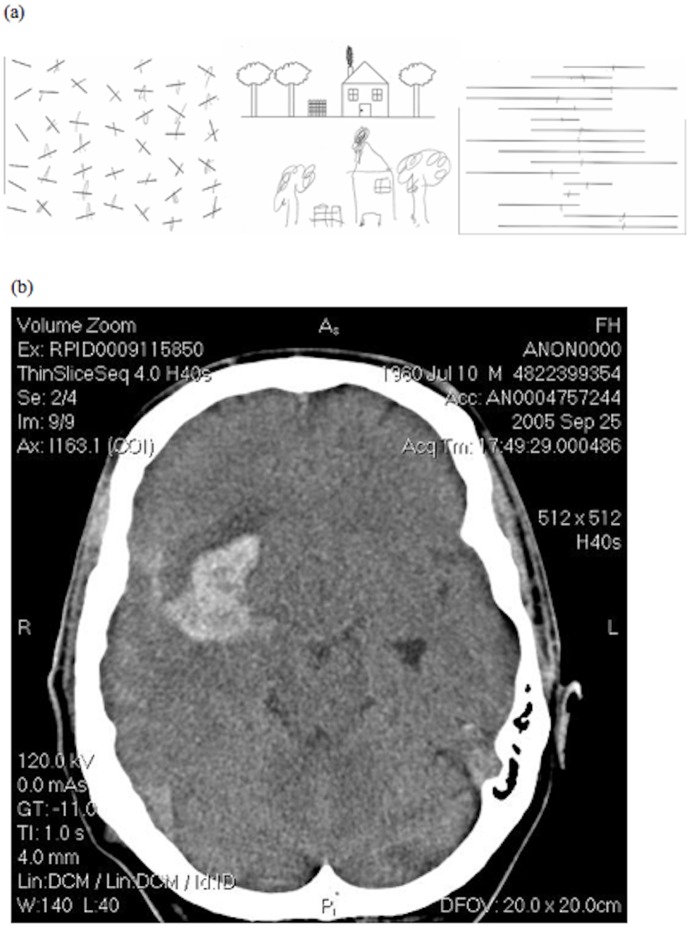
(a) shows some of P1’s screening data for the line bisection, cancellation and scene copying tasks. (b) A CT scan of P1 showing the haematoma with bleeding, and the dark area anterior to it, which is the haemorrhagic stroke.

At the time of testing P1 was a 46-year-old left-handed male who had suffered a stroke two months prior to recruitment, having previously worked as a museum attendant. A CT scan (see Figure 1 legend) taken shortly after hospital admittance showed a large (4 cm) intracerebral haematoma with haemorrhagic stroke in the temporoparietal region of the right hemisphere. Screening of P1 was carried out six months post-stroke using a battery of subtests from the Behavioural Inattention Test [17] including cancellation tasks, line bisection tasks and scene copying that all showed that P1 neglected information presented in his left hemifield. Figure 1(a) shows some of P1’s screening data, see Figure 1 legend.

P1 acted as a voluntary unpaid participant in the following experiments. He had corrected to normal vision and was naïve in relation to the purpose of the investigation. Ethical approval was obtained from the Newcastle Upon Tyne Hospitals NHS Trust and written informed consent was obtained from P1 and control participants.

## Experiment 1: Verbal Detection Task

The aim of the verbal detection task was to test how long stimuli had to be presented in P1’s visual fields before he became consciously aware of their presence. Simultaneous presentation of one stimulus in each hemifield can result in detection of only the stimulus presented in the right visual field (e.g. [18]) and is known as extinction.

### Method

#### Materials

Targets and distractors were small black solid squares, approximately 0.6 degrees in size, set against a grey background. The centres of targets and distractors were positioned at 5 degrees to the left or right of the midline of the display. A display with a black central fixation cross was created for inter trial presentation to facilitate central fixation at the beginning of each trial.

#### Design

A repeated-measures design with independent variables of Target Type (single left, single right and bilateral targets), and Target Duration (50 ms, 100 ms, 200 ms, 400 ms, 800 ms, 1000 ms, 2000 ms, 3000 ms, 4000 ms and 5000 ms) and dependent variable of response accuracy was employed. P1 completed one block of 180 trials in which a single target could appear either on the right (56 trials, 8 per each duration) or the left (56 trials, 8 per each duration) of the screen, or, in the bilateral condition, two targets appeared, one on each side of the display (56 trials, 8 per each duration). Random presentation of the target types occurred during the block along with 12 catch trials where no targets appeared.

#### Procedure

P1 was positioned with his eyes in line with the middle of the screen at a viewing distance of 57 cm. Following each trial P1 had to verbally report what he had ‘seen’ by responding, ‘one’, ‘two’ or ‘none’, and was aware that if he reported ‘two’ this indicated that there was a target present on each side of the display. Each trial was initiated following P1’s verbal response to the previous trial.

### Results and Discussion

**Figure 2:**
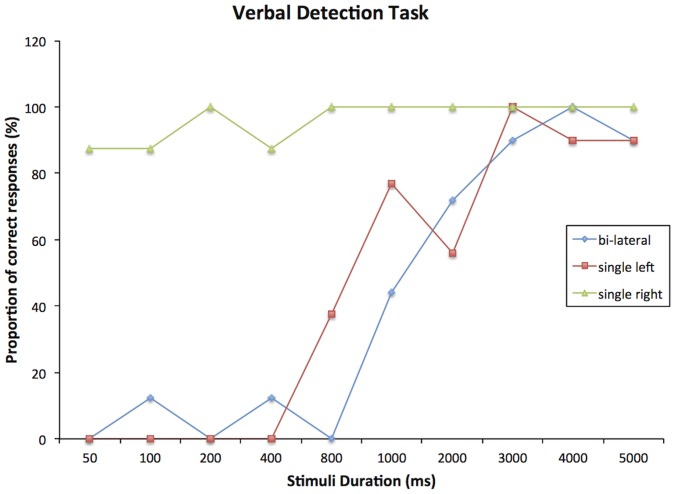
Shows the proportion of correct responses for the different types of target (single right or left or bi-lateral) against the time each of these were presented for.

P1 responded ‘none’ on 100% of the catch trials, showing that he was not simply guessing when making his responses. On target present trials, at presentation durations of 1 s P1 detected 100% of single right targets, 77% of single left targets, and 44% of bilateral targets (reported both left and right). At durations of 3 s P1 detected 100% of single left targets and 90% of bilateral targets. At durations of 400 ms and below, P1 detected 100% of single right targets, no single left targets, and no bilateral targets. The verbal detection task confirmed that P1 was unaware of single left targets when these were presented for less than 400 ms. At 800 ms his detection rate for single left targets was still under 50%. During bilateral presentation he reported right targets only at durations of 800 ms and above. P1 therefore presented with a clear neglect, while the additional effect of extinction was quite small. See Figure 2 legend, the figure shows P1’s responses on the verbal detection task. These data were used in the next experiment to examine whether stimuli presented in P1’s neglected hemifield had to be presented at durations at which he showed some awareness for in the verbal detection task, in order to program an eye movement to those targets.

## Experiment 2: The Remote Distractor Task

The Remote Distractor Effect (RDE) is a robust low-level visual effect whereby an increase in saccade onset latency (20–40 ms) occurs when two possible targets are presented simultaneously (rather than only one), and the task is to saccade to one of these targets [19]. The RDE has been assumed to be an automatic reflexive property of the saccadic system [20] and is believed to result from activation in the fixation cells in the superior colliculus [19]. Assessment of the RDE in P1 would show whether low-level interactions in the saccadic system were normal for the presentation of contralesional stimuli. Although some work has been conducted in this area (e.g. [8]; [21]), surprisingly the studies have not attempted to manipulate attentional orienting. Using the RDE paradigm we examined the effects of the prior allocation of attention to either ipsilesional or contralesional space on saccadic orienting in P1, and also evaluated whether there was any dissociation between the saccadic orienting system and conscious awareness (as indexed by the verbal detection task) for stimuli presented in P1’s ‘neglected’ hemifield.

### Method

#### Eye movement recording

For the following experiments eye movements were monitored using an *Eyelink 1* video-based eye tracker with spatial gaze resolution of <0.5 degree. P1’s chin was positioned in a chin cup to facilitate stable fixation and minimise gross head or body movements during testing. Eye position was sampled every 4 ms. Viewing was binocular, but only the movements of the right eye were used for analyses. Stimulus files were displayed on a 19-inch monitor at a viewing distance of 57 cm. Calibration was carried out by asking P1 to sequentially fixate points on the display that covered the appropriate dimensions for each relevant task. To calibrate in the neglected field P1 followed the experimenter’s finger to each point and remained fixating it until asked to move his eyes to the next one.

#### Materials

The same stimulus displays employed in the Verbal Detection task were used for the RDE experiment.

#### Design

A repeated measures design was employed with independent variables of Target Type (single left, single right and bilateral targets), and Instruction for bilateral target presentation (go left, go right, go to either, whenever two targets were presented simultaneously). Dependent variables were saccade onset latencies (the time taken to initiate a saccade from the onset of the trial display) and directional errors (eye movements directed to the target in the opposite direction to the task instruction). P1 completed three blocks of 180 trials in which a single target could appear either on the right (60 trials), on the left (60 trials) of the screen, or, in the bilateral condition, the target appeared with a peripheral distractor positioned mirror symmetrically in the opposite hemifield to the target (60 trials). Random presentation of the target types occurred for all blocks.

#### Procedure

P1 was instructed to move his eyes to the target as quickly and accurately as possible whenever a single target was presented. In the case of two possible targets appearing P1 was instructed as to which target to move his eyes to prior to each block of trials. Each trial began with a central fixation cross for a variable d of either 500 ms, 1000 ms or 1500 ms and simultaneous with the offset of this a target was presented for 1s, either in isolation or with a distractor in the opposite hemifield, followed by a blank screen for 1 s. We knew (from the verbal detection task) that at this presentation duration P1 had some awareness for targets presented in his neglected hemifield (70%) and for bilateral targets (44%). A practice block (20 trials) preceded experimental blocks with breaks taken on a request basis. In block A, P1 was instructed to look to either target when two targets were presented; in block B, P1 was instructed to look to the right target when two targets appeared; and in block C, P1 was instructed to look to the left target when two targets were presented.

### Results and Discussion

Eye movement onset latency was detected automatically using a velocity criterion of 30deg/s and each record was inspected individually. Trials were removed (29% total) if signal loss occurred (9%), saccades started before 100 ms (11%), the eye was off-centre (9%) at the onset of stimulus display.

#### Error data

**Figure 3:**
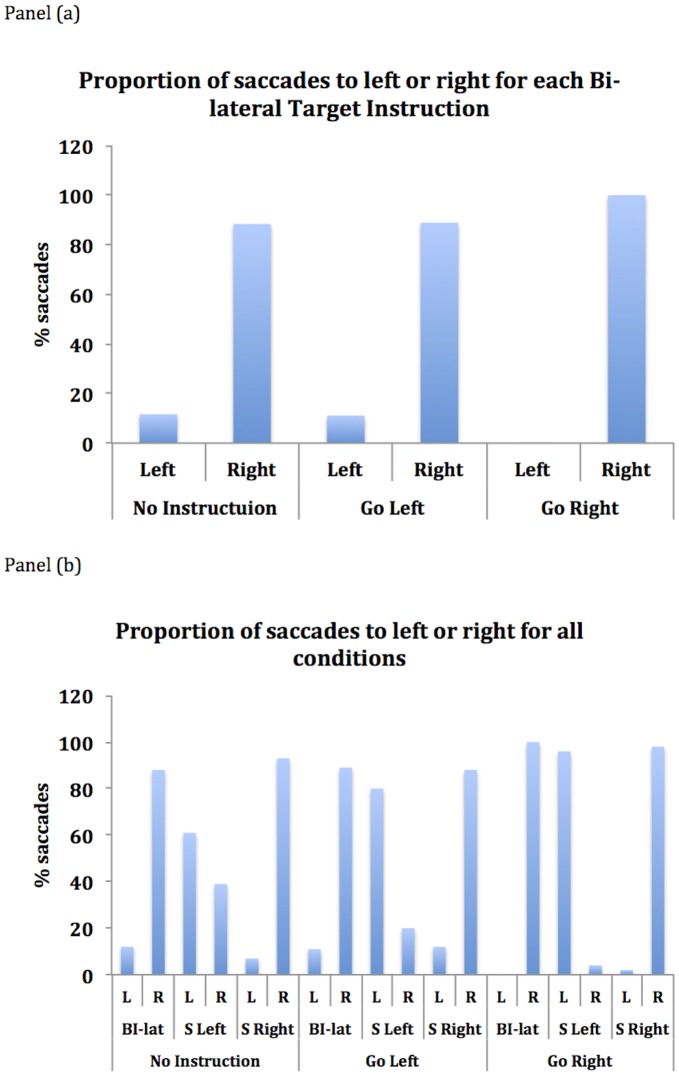
Shows the proportions of saccades directed to left and right for the different conditions. Panel (a) shows the proportion of saccades to left and right for each bi-lateral target instruction and panel (b) shows the proportion of saccades to left and right for all conditions.

Directional errors (19%) were excluded from the analysis of eye movement onset latency. See Figure 3 legend. The figure shows the proportion of saccades directed to the right or the left for each of the three bi-lateral target instructions, and the proportions of left and right saccades for all conditions, and illustrates the error data. When P1 was instructed to go left on bilateral target presentation he was unable to do this and instead moved his eyes to the distractor on the right on 89% trials. Additionally, in the condition where he was free to go to either possible target, P1 moved his eyes to the right target on 88% of trials. These data show that the attentional bias to the right visual field shown by P1 appears not to be under voluntary control and are similar to Van der Stigchel and Nijboer [12] who used a variant of the RDE paradigm to examine oculomotor capture in neglect. The error data support the theory that neglect involves hyperattention to the ipsilesional field (e.g. [10]) rather than hypoattention to the contralesional field [11].

#### Saccade latency analyses

Saccade latency was compared in a two factor ANOVA with Target Type (single left, single right, bilateral) and Instruction for the bilateral presentation (go left, go right, go to either) as independent variables. A main effect was observed for Instruction (F (2,315) = 10.70, p<.0001), which showed that latencies were longer for the look-left condition (272 ms) compared to the look-right condition (220 ms) and the uninstructed condition (208 ms). A main effect was also observed for Target Type (*F* (2,315) = 59.10, *p*<.0001), which showed that latencies were longer for the single left targets (313 ms) compared to those for single right targets (187 ms) and saccades generated for bilateral target presentation (200 ms). There was no interaction between Instruction and Target Type (*F* (4,315) = 1.40, *p*>.24). Table 1 shows the mean eye movement latencies and the standard deviations for each condition. P1 showed a pattern of overall slower saccades to single left targets, which has also been recently demonstrated in another patient [12], and slower saccade execution in the condition where he was instructed to saccade to the left target when two targets were presented simultaneously.

**Table 1:** The mean onset latencies and standard deviations (in parentheses) for each condition for the RDE Experiment.

Mean saccade latencies for correct responses (ms)					
Instruction for bilateral targets			Target Type		
Go to either	Go left	Go right	Single Left	Single Right	Bilateral
208 (86.95)	272 (125.48)	220 (90.04)	313 (114.24)	187 (66.41)	200 (75.41)

#### RDE comparisons

**Figure 4:**
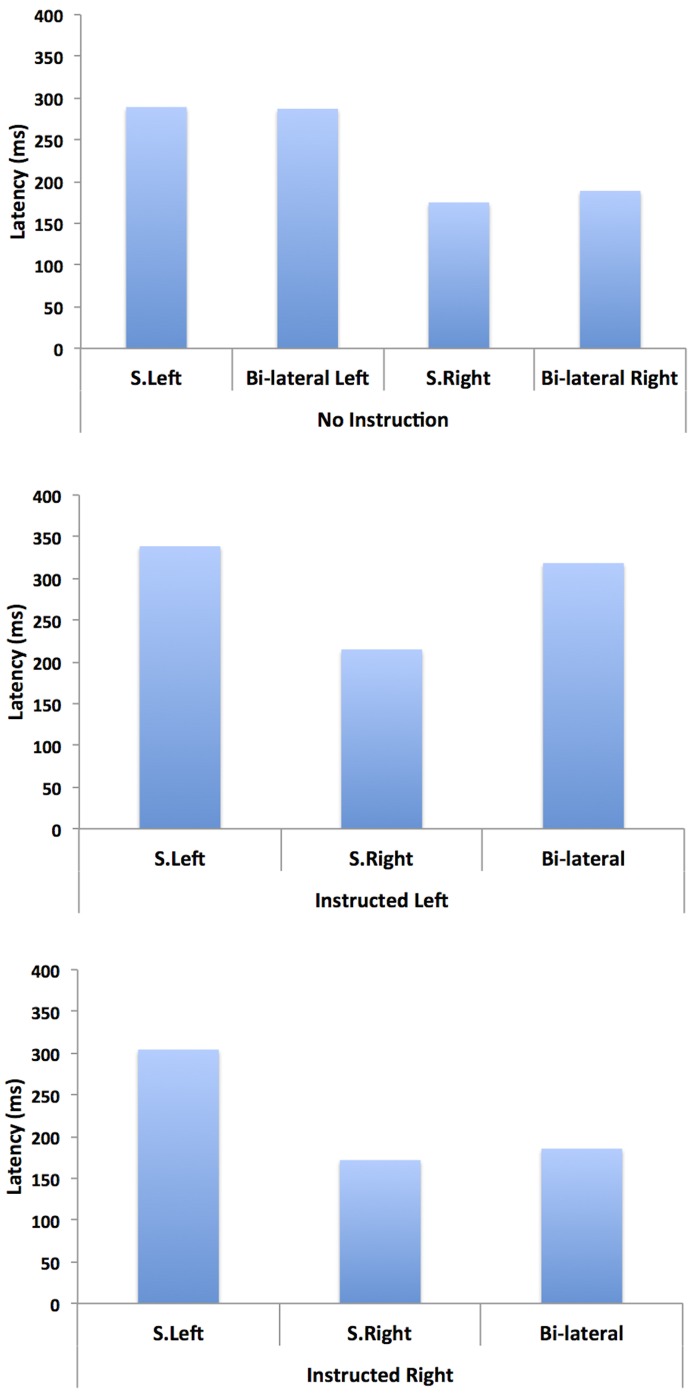
Shows the mean latency for eye movements executed in each of the bi-lateral target instruction conditions, for single right and left saccades, and for saccades that were made when two possible targets were presented.

To check whether P1 showed evidence of implicit processing for stimuli presented in his contralateral hemifield we compared the latency for the single target trials to the latency for trials where a distractor was presented with a target, for all conditions (see Figure 4 for the means). In the condition where P1 was free to move to either target in bilateral presentation, comparisons were made separately for bilateral trials where he looked toward the right target and where he looked toward the left target.

In the No Instruction condition single target-right latencies were not significantly different from latencies to the right target in bilateral presentation (*F* (1, 79) = 1.27, p = .264), and single target-left latencies were not significantly different from latencies to the left target in bilateral presentation (*F* (1,25)<1). In the Instructed right condition, there was no significant difference between latencies for single right targets, and latencies for right targets when a distractor was present in the left hemifield (*F* (1,86) = 1.196, p = .277). Finally, in the Instructed left condition, there was no significant difference between latencies for single left targets, and latencies to left targets when a remote distractor was present in the right hemifield (*F* (1,38)<1), though the error data showed that there were only a small number of trials where P1 was able to do this making this comparison unreliable. Together, these analyses suggest that independent of instruction type and saccade direction, P1 showed no evidence of implicit processing of distractors presented simultaneously with a saccade target.

P1 did not show the characteristic RDE effect when distractors were presented in his contralesional hemifield, a finding consistent with previous reports (e.g. [8]). The lack of a RDE in hemi-neglect has been proposed to reflect the absence of competition between fixation-neuron activity in the two superior colliculi that normally occurs when two rather than one possible targets are presented simultaneously [12]. Since the SC receives extensive direct projections from the cerebral cortex on the same side of the brain [22]–[23], large cortical lesions could be expected to deprive the ipsilateral SC of substantial downstream facilitation effects. It is important to note here that in the verbal detection task P1 reported awareness for bilateral targets presented in his left field for durations of 1s on 44% of trials. Therefore, the failure to observe a RDE in P1 occurred even though he would have been consciously aware of the distractor in his neglected field on almost half of the trials suggesting the possibility of a dissociation between awareness for bilateral targets and P1’s saccadic orienting system. The earlier work on the RDE in neglect has assumed unawareness for distractors in the neglected field, hence explanations for the lack of an RDE were thought to reflect an imbalance in the saccadic system [8], but here we have shown a failure to elicit an RDE in the presence of awareness for distractors. We think that our findings do not reflect a simple imbalance in the saccadic orienting system per se; rather, we think that the hyperattention to the right shown by P1 (from our error data in this task) is also responsible for the lack of an RDE.

In the verbal detection task P1 failed to report the presence of single left targets in his contralesional hemifield when these were presented at durations of 400 ms, however, in the RDE task eye movement recordings demonstrated that he was able to make saccades to single left targets on approximately 70% of those trials, and that the average latency for those saccades was 313 ms. This means that P1 is capable of moving his eyes to targets for which he shows no awareness for in the verbal detection task. Thus, we think our findings suggest that the relationship between awareness and saccadic orienting is disrupted for both the ipsilateral, and the contralateral fields in P1 for low-level orienting tasks. Furthermore in higher-level cognitive processing tasks, described below we were also able to contrast awareness and eye-movement patterns within the same tasks.

## Experiment 3: Scanning of a Complex Visual Scene

Patterns of saccades and fixations can reflect not only which features of a display drive the eye movements for a given task [5] but also how task instruction can modulate saccadic scanning for the same stimulus. For example, the early work of Yarbus [24] provided a compelling demonstration of how eye movements can reflect the on-line cognitive processing during complex scene inspection in healthy individuals. We used a modified version of the Yarbus study [24] to investigate whether P1 showed evidence of an ability to modulate his voluntary saccadic orienting in line with different inspection instructions during complex scene viewing.

### Method

#### Materials and procedure

The Repin picture was downloaded from (http://www.abcgallery.com), and presented to P1 seven times, each presentation lasting 20 seconds. The same inspection instructions as those used in the Yarbus study were given to P1 on a trial by trial basis, and, in addition, the picture was flipped in the horizontal plane such that the ‘unexpected visitor’ appeared on the left for half of the trials, and on the right of the picture for the other half of the presentations. P1 was informed of this manipulation and was shown the picture in both orientations prior to testing. Two additional inspections of the Repin picture were also completed. In trial 8 the instruction was to count the number of people in the picture. In trial 9 a previous instruction was repeated, but the orientation of the picture was switched such that the ‘unexpected visitor’ was on the opposite side to the original presentation. Following each inspection P1 gave a verbal response and a transcript of these, along with order of inspection instruction, and a record of the location of a critical figure in each scene presentation is shown in table format in the appendices (see Appendix S1). This information is also given for a control participant (C1) with right brain damage and no neglect. At the time of testing C1, a 61-year-old male had suffered a first stroke three months previously. A CT scan revealed damage to the right fronto-parietal region. Initial screening showed minor evidence of neglect for drawings which recovered and which was absent at testing for the Repin study.

### Results and Discussion

#### Proportion of fixations and dwell time

**Figure 5:**
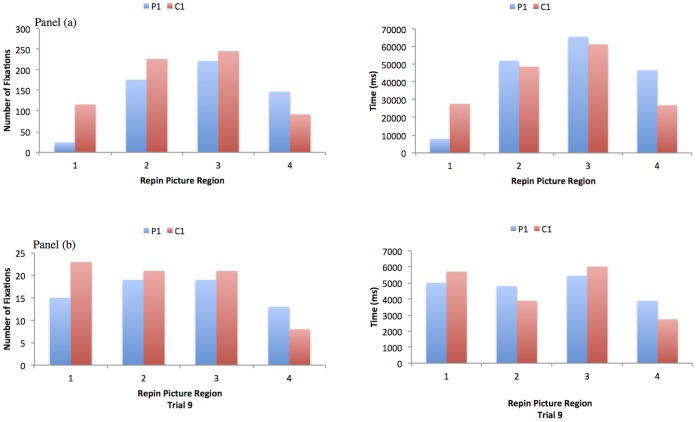
Shows the distribution of fixations and proportion of time, for the two participants, across the different regions of the Repin picture. Panel (a) shows the amount of time and the number of fixations made to the four different regions of the Repin picture over the first eight inspection instructions, for P1, and for a control participant C1. Panel (b) shows the amount of time and the number of fixations made to the four different regions of the Repin picture for the final trial where one of the inspection instructions was repeated.

Vertical calibration problems made it impossible to analyse the exact distribution of fixations to specific objects in the scene on all trials. Therefore sampling biases in P1 were examined by dividing the Repin picture into four equal sections, far left (Region 1), left (Region 2), right (Region 3) and far right (Region 4), where left and right refer to the side of space from the midline of the picture, and comparing the mean number of saccades made to, and the mean total time spent in each region, , see Figure 5 legend. Overall, the far left region of the Repin picture was sampled less often and for less time in P1 compared to the other regions, and compared to C1.

#### Scanpaths

Plots of P1's scan paths during each inspection (see Appendix S1) showed that he was able to scan and fixate both sides of the picture, although there were occasions where he failed to fixate relevant items/people in the scene. However, comparing proportions of fixations and time spent in either half, or in smaller sections of the picture says little about how the picture was sampled over time.

#### Temporal analyses

A temporal analyses showed that although P1 was able to scan and fixate the left half of the scene, this was never done in an immediate way. For example, during a free viewing inspection of the scene, P1 scanned the right half of the picture for over 10 seconds before he began inspecting the ‘unexpected visitor’ who was positioned on the left of the scene in that trial. By contrast, C1 fixated this critical figure in the same position and under the same inspection instruction in less than 2 seconds. For another of the instructions the display was presented in one orientation, and later, it was presented again in the opposite orientation. For these two presentations P1 and C1 were asked to “estimate how long they thought the ‘unexpected visitor’ had been absent for". A qualitative difference between P1 and C1 in the pattern of saccades was observed for each viewing, and a temporal analysis of the saccades and fixations made during each trial display showed that regardless of orientation, C1 fixated the ‘unexpected visitor’ in less than 2 seconds, whereas P1 fixated the ‘unexpected visitor’ within 2 seconds when he appeared on the right of the display (first presentation) but took over 8 seconds to fixate him when he appeared on the left, see Figure 5 legend.

Thus, despite consciously knowing the location of the critical information based on previous alternating picture orientations P1 is unable to move to that information in the scene immediately. Furthermore, for the inspection instruction ‘count the number of people in the picture’ P1 reported that there were 12 people, where in fact there were only 7. During this inspection P1 failed to fixate the two children positioned on the far left, and so one, tentative, explanation is that he multiplied the number of people that he did fixate by two, and then added the two children that he had ‘seen’ when they were presented in a previous orientation on the far right of the picture. Movie clips of trials 7, 8 and 9 are in Appendix S3 for P1.

This study has shown that P1 cannot easily modulate his voluntary saccadic orienting in line with different inspection instructions during complex scene viewing, and furthermore, the data also reveal that for this high level cognitive task there is also a dissociation between what P1 looks at (sampling) and what he sees (awareness).

## Experiment 4: Text Reading

Neglect dyslexia describes errors made in reading where either whole words or the left part of words are misread or omitted [25] and hemi-neglect can result in this condition. Anecdotally, P1 has reported that he is an avid reader of novels, but given that he neglects information on the left, how is he able to accomplish this task? We investigated whether P1 exhibited a normal pattern of saccades for reading, and if not, whether he could nonetheless comprehend what he read. Only one paper has examined eye movements and text reading in neglect [26], and our experiment provides the first eye-movement study of single sentence reading in neglect to investigate whether scanning of the text corresponds with comprehension.

### Method

#### Materials and procedure

We presented P1 with 20 sentences, none of which exceeded a single line in length, one at a time, positioned in the centre of a computer screen. P1 had to read each sentence silently whilst his eye movements were recorded, and following each sentence a comprehension question was asked. The information needed to answer the question was positioned on the far left of the screen (at the beginning of the sentence) for half the sentences, and on the far right of the display (at the end of the sentence) for the other ten sentences. See Appendix S2 for the sentences presented paired with the relevant comprehension question, and the transcribed response for P1.

### Results and Discussion

P1 made no comprehension errors when the relevant information appeared towards the end of the sentence, but was correct on only 50% of occasions when the relevant information was presented in his neglected field. Based on the semantic content of P1’s incorrect answers, it appears that he made inferences related to those portions of the sentence he had actually read. For example, the following sentence ‘The large tiger lay in the sun and ignored the tourists taking photos’ was followed by the question ‘What ignored the tourists’? and P1 responded ‘people lying in the sun’.

In the analyses of the eye movement data, as is standard in reading studies (e.g., [4]), fixations lasting less than 80 ms and greater than 800 ms were removed. Below we provide a broad description of several eye movement measures where P1 differs significantly from an age matched healthy control participant (C2). Paired t-tests showed that the mean number of saccades from left to right during sentence reading was greater for C2 (mean 10.7) compared to P1 (mean 8.15); (t (1, 38) = 5.00; p<.001), whereas the mean number of saccades from right to left during reading was greater for P1 (mean 5.20) compared to C2 (mean 1.25), (t (1, 38) = 10.20; p<.001). Furthermore, the range in words (1–15) over which P1 made right-to-left fixation shifts was reduced (mean 9.85), relative to C2 (mean 13.30); (t (1, 38) = −8.36; p<.001). Clearly, P1’s saccadic movements during reading were markedly different to those of the control participant, who showed a normal reading pattern [27]. This finding might initially seem somewhat counterintuitive, in that P1 made more leftward saccades, into his neglected field, than the control participant. However, the fact that P1 made so many leftward movements can explain why he did not exhibit the typical sequence of left to right saccades during reading. P1 made significantly more regressive movements back through the sentence (mean 4.75), compared to C2 (mean 1.25); (t (1, 38) = 9.89; p<.001) and these regressions appear to have been made for two reasons; first, in order to re-inspect text that he had already fixated, and, secondly, to allow him to identify a point towards the beginning of the sentence (e.g., a clause or phrase beginning) that could potentially be the first word of each sentence. Indeed, P1 was never able to locate the word at the beginning of each sentence with his first fixation (P1 0%) compared to the control (C2 100%), and on average across the sentences, his first fixation landed on the fourth word (s.d. = 1.5) of the sentence.

We also examined characteristics of P1’s fixation durations and reading times in relation to the control participant. P1 had significantly longer total reading times (mean 4.5s) for the sentence than C2 (mean 3.2s) (t (1, 38) = 6.80; p<.001), however, his average fixation duration during reading (mean 242 ms) did not differ from that of C2 (mean 244), (t<1). Durations of fixations during reading are largely determined by the ease with which a word can be identified [3]–[4]. Thus, the average fixation duration data suggest that P1 was perfectly able to lexically identify each of the individual words of a sentence when he fixated them. Together, the eye movement data suggest that the primary source of difficulty was reading the text from left to right sequentially, i.e. processing the words in their appropriate order, making it unlikely that processing difficulty was linguistic in nature.

Word skipping usually occurs for short high frequency words within the language, however, given that P1 often made many regressive saccades as he searched for a leftward point in the sentence from which to start reading, his skipping behaviour does not resemble that observed in a typical reader. On average, P1 skipped 4.8 words in the sentence, as contrasted with 1.2 words skipped by C2 (t (1, 38) = 9.35; p<.001). In other words, P1 failed to directly fixate approximately a third of the words within a sentence, contributing to his comparatively low comprehension scores. We also computed the number of words P1 revisited, along with the number of re-fixations he made on words overall (either after a fixation on the same word, or after a fixation on a different word). P1 made many more revisits to words (mean 4.8) than C2 (mean 1.3), (t (1, 38) = 9.90; p<.001), and many more re-fixations on words (mean 9.7) than C2 (mean 1.6), (t (1, 38) = 12.40; p<.001). Once again, the increased revisits and re-fixations that P1 made as he read the sentence are entirely consistent with the conclusion that he experienced significant disruption to cognitive processing as he read the sentences. A similar tendency to re-visit items is commonly seen in visuospatial search tasks in neglect patients [28]. Table 2 shows the means and standard deviations for the reading measures for both participants.

**Table 2:** The means and standard deviations for the reading measures for P1 and the control participant C2.

Variable	Participant	Mean	Std. Deviation
Number of fixations	P1	18.6	(2.09)
	C2	13.4	(2.66)
Total time (ms)	P1	4500.	(447.21)
	C2	3245.	(699.34)
Mean fixation duration (ms)	P1	242	(20.51)
	C2	244	(37.28)
Number of words skipped	P1	4.75	(1.59)
	C2	1.15	(0.67)
Rightward saccades	P1	8.15	(1.95)
	C2	10.7	(1.17)
Leftward saccades	P1	5.2	(1.67)
	C2	1.25	(0.44)
Number of words refixated	P1	4.75	(1.02)
	C2	1.25	(1.21)
Number of refixations	P1	9.7	(2.45)
	C2	1.55	(1.64)
Fixation range	P1	9.85	(1.69)
	C2	13.3	(0.73)

In normal populations readers systematically make a series of left-to-right saccades from a starting fixation on the leftmost word in the sentence until they fixate the final word in the sentence. Although leftward saccades do occur, these are usually associated with words or phrases that cause linguistic processing difficulty and the overall pattern of saccades and fixations is influenced by a variety of visual and linguistic variables (e.g. [4]). P1’s abnormal pattern of saccadic orienting during reading shows that he is not able to move his eyes using the optimal strategy; instead, in an attempt to comprehend what he is reading, he makes repetitive regressive saccades. Movie clips of P1 and C2 recorded while reading one of the sentences where P1 answered the comprehension question incorrectly are provided in Appendix S3.

When P1 was asked to read a further twenty sentences out loud, his accuracy was 50%. In the incorrectly read sentences P1 made left word omissions (10%), mispronounced the left portions of one of the words (5%), added words to the sentences (30%), and failed to complete the sentences (10%). There were also long gaps, either before beginning to read the sentences, or sometimes during the reading of them. Together the reading data suggests that P1 is unable to sequentially fixate the words in a sentence, and instead he appears to rely on holding individual words in memory temporarily before mentally arranging them into something that approximates a meaningful sentential interpretation, which he then ‘reads out. In line with the two studies reported above, the consistent finding from P1 is of a disjoint between what he is looking at and what he actually perceives.

## General Discussion

An important question concerning neglect relates to whether an inability to ‘see’ something results from a failure to ‘look’ at that something, which would indicate a deficit in saccadic orienting, or whether information is ‘looked’ at but still not actually ‘seen’, which would indicate a deficit at a higher level of perceptual processing. We have tried to address these issues in this paper by examining eye movements and verbal responses for both low-level oculomotor control tasks and higher-level visuo-cognitive processing tasks.

It appears in the case of P1 that his low level reflexive orienting system is intact, at least as shown by his ability in the RDE paradigm to orient to single targets presented in his neglected field, albeit with a slower latency than he is able to orient to targets in his good hemifield. However he does not show the characteristic RDE, and appears often not to ‘see’ the targets that he has looked at as shown by his responses in the verbal detection task, demonstrating a dissociation between awareness for stimuli and an ability to orient to those stimuli reflexively in this very simple task. The scene inspection findings reflect an inability in P1 to voluntarily ‘look’ to information presented in his neglected field, which he can overcome if he is made aware that crucial information is presented there, but his default strategy is to initially orient to information in his right visual field. Moreover, in this higher -level task the sequence of eye movements should be cognitively driven, and this is not the case for P1, who is unable to accurately report information that has been looked at. Finally P1’s eye movements for reading single sentences also show disordered scanning coupled with an inability to correctly report visually sampled information in his neglected field. Together the findings indicate that in P1 there is a dissociation between the eye movement sampling system and conscious processing of information that has been sampled, lending support to the notion that in neglect the defining characteristic is not simply an imbalance in the saccadic orienting system, as shown by the RDE study, but also an inability to perceive information that has been sampled, as shown by the scene inspection and reading studies.

Our findings from the P1 experiments do not fully support Heilman’s [1] original definition of neglect, as the “failure to respond to, orient towards, or report, stimuli located on the side of space contralesional to brain damage in the absence of sensory motor deficits". In particular, the failure to report a stimulus in the neglected field is not necessarily coupled with a failure to fixate that stimulus. Our data fit with recent findings showing that rehabilitation techniques that ‘force’ low-level oculomotor shifts of attention into the neglected field, e.g. prism adaptation [29] which may affect dorsal rather than ventral pathways [30], fail to result in any real improvement in perceptual awareness in neglect. As we have shown with P1, a shift in gaze does not necessarily equate to a perceptual shift.

In the introduction we proposed that the evidence from eye movement studies had not conclusively demonstrated whether neglect reflects a failure to look at (sample) relevant information, or a failure to show awareness for (process) relevant information in the neglected field. What appears to be disrupted in P1 is the tight coupling that is normally shown between attention and eye movements. For example, one can move attention to a new location without moving the eyes (e.g. [31]), but it has been argued that in normal populations moving the eyes to a new location is usually accompanied or preceded by a shift in attention (e.g. [32]) to that location, when engaged in a task. This is not the case for P1, and this impaired relationship between attention and eye movements could account for P1’s ability to ‘look’ at information without ‘seeing’ it. Future work with larger samples should aim to test, experimentally, whether the lack of awareness for stimuli in the neglected field results from disruption between the eye movement orienting system and the attentional orienting system. These two systems may be subserved by the same neural circuitry (e.g. [33]) in normal populations, and they normally work together in the visual selection process.

## Supporting Information

Appendix S1 **Shows a transcript of the verbal responses for both participants along with order of inspection instruction, a record of the location of a critical figure in each presentation and the participant’s eye movements overlaid for each inspection.**
(DOCX)

Appendix S2 **Shows the sentences presented, paired with the relevant comprehension question, and the transcribed response for P1.**
(DOCX)

Appendix S3 **Includes some movie clips for some trials from the scene inspection and reading studies.**
(DOCX)
