# Supplementary material for: Eye Movements and Verbal Report in a Single Case of Visual Neglect
Source: PLoS One. 2012 Aug 24;7(8):e43743. doi: 10.1371/journal.pone.0043743 (PMC3427146; doi:10.1371/journal.pone.0043743)
Supplement: Appendix S1 — Shows a transcript of the verbal responses for both participants along with order of inspection instruction, a record of the location of a critical figure in each presentation and the participant’s eye movements overlaid for each inspection. (DOCX) [file pone.0043743.s001.docx]

Appendix S1: Saccades and Fixations for the Repin Task – see Yarbus [24] for original scanpaths for these inspection instructions.

| Task | C1 - Control - response | P1 - Neglect - response |
| --- | --- | --- |
| 1. Free viewing |  |  |
|  | 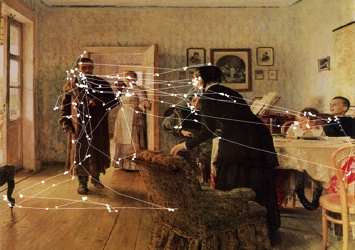 | 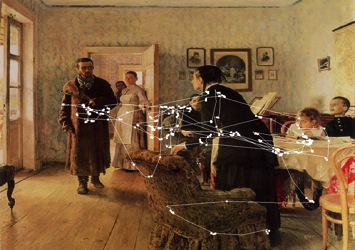 |
|  | | |
| 2. Estimate the material circumstances of the family | Well off | Fairly wealthy |
|  | 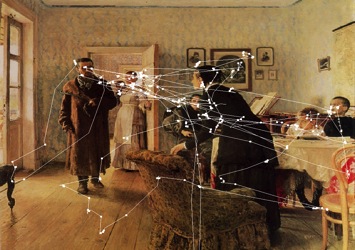 | 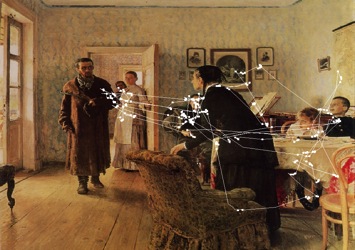 |
|  | | |
| 3. Give the ages of the people | Guy 50’s  2 children to the left, 6 & 7  Lady at door 40’s… | Woman in black - late 50’s  Man in brown coat – late 40’s |
|  | 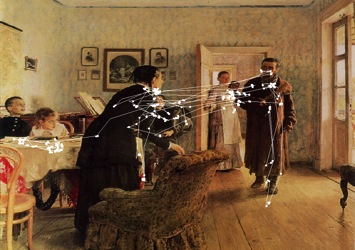 | 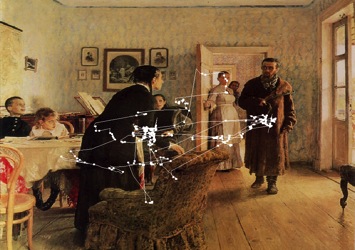 |
|  | | |
| 4. What were the family doing prior to the arrival of the visitor? | Having a musical time or singing | Eating – having a meal |
|  | 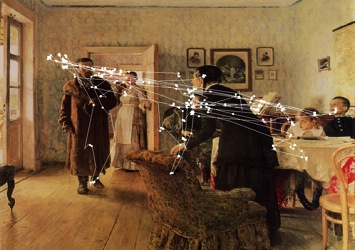 | 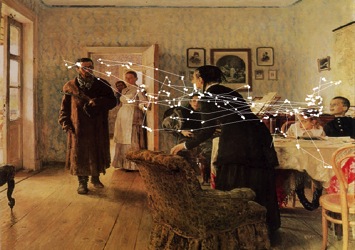 |
|  | | |
| 5. Remember the clothes worn by the people | Man entering room is in outdoor clothing, lady dressed in black, lady at door looks like a cook, wearing a pinny…. | Woman in black wearing mourning clothes  Guy – outdoor coat |
|  | 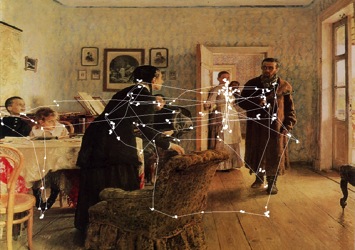 | 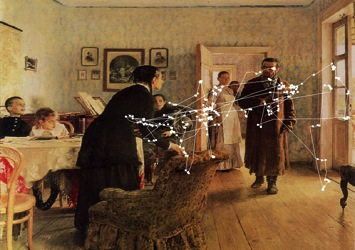 |
|  | | |
| 6. Remembers the positions of the people and objects in the room | Armchair – lady in black resting upon it, 2 children sat at a table, maybe a piano in the background…  Mentioned all the people and other objects | 2 young children at the table  2 servants in the open door  Woman in black by the armchair  **No mention of man** |
|  | 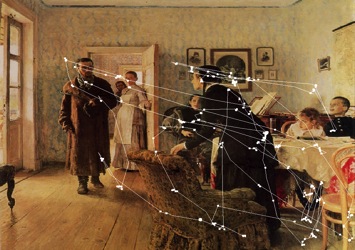 | 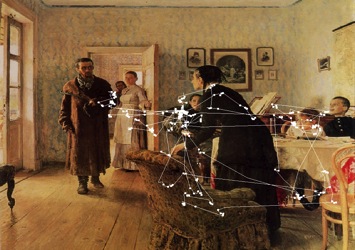 |
|  | | |
| 7. Estimate how long the visitor had been away from the family. | days | No idea – nothing in the picture to pick up on to tell that information |
|  | 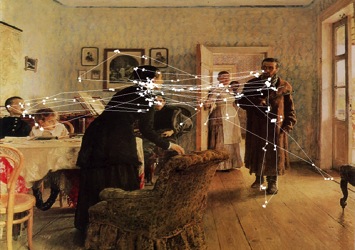 | 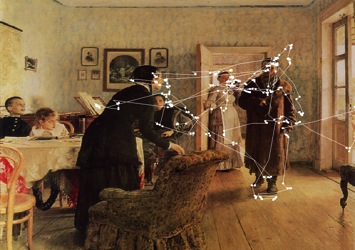 |
|  | | |
| 8. Count the number of people in the picture. | Seven people | 12 people in the picture – ‘name them’  2 servants doorway  Guy in brown coat  Woman of the house in black  2 kids at the table  Young girl  Older child, That’s it |
|  | 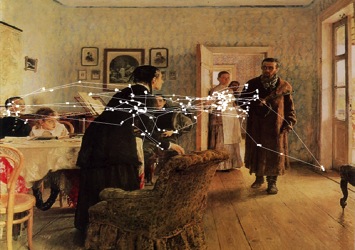 | 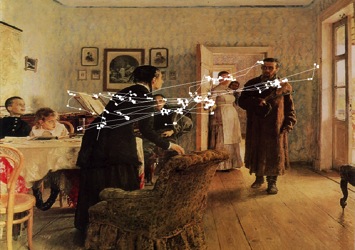 |
| **BREAK** | | |
| 9. Estimate how long the visitor had been away from the family. | days | On pressing for an estimate - Family look fairly surprised so at least days, but don’t really know |
|  | 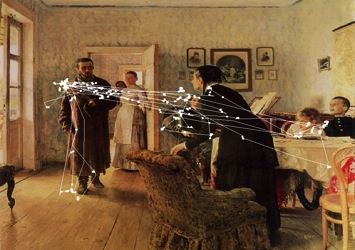 | 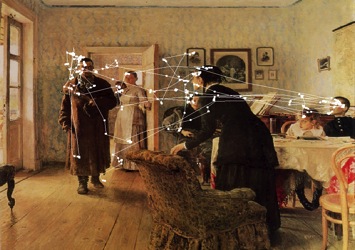 |
