# Supplementary material for: Eye Movements and Verbal Report in a Single Case of Visual Neglect
Source: PLoS One. 2012 Aug 24;7(8):e43743. doi: 10.1371/journal.pone.0043743 (PMC3427146; doi:10.1371/journal.pone.0043743)
Supplement: Appendix S2 — Shows the sentences presented, paired with the relevant comprehension question, and the transcribed response for P1. (DOCX) [file pone.0043743.s002.docx]

Appendix S2: Sentences, Comprehension Questions and responses for the Reading Study.

Information on the left

1. On Tuesday John went to the flower shop to buy a large bunch of daffodils.

Q. On what day did John buy the daffodils?

Response: ‘Tuesday, was that Tuesday?’

2. Jane often visited her aunty Margaret early on a Sunday afternoon for tea.

Q. Who visited Aunty Margaret on a Sunday afternoon?

Response: ‘Jane’

3. For three days the storm had lasted and the heavy rain had caused floods.

Q. How many days did the storm last for?

Response: ‘3 days’.

4. The pink car attracted a lot of attention amongst the shoppers in the town.

Q. What attracted attention in the town?

Response: ‘the car’.

5. The large tiger lay in the sun and ignored the tourists taking photos.

Q. What ignored the tourists?

Response: ‘people lying in the sun’.

6. The yacht looked fantastic and would cost more money than Tom could afford.

Q. What was too expensive for Tom?

Response: ‘the yacht’.

7. A month later they decided that something must be done about the problem.

Q. How long was it before they decided to solve the problem?

Response:’ not sure’.

8. The dog was very dangerous and the young children were very scared of it.

Q. What scared the young children?

Response: ‘dog’.

9. The fireman ran round the corner unaware of the problems he would encounter.

Q. Who ran round the corner?

Response: ‘the man’.

10. The book fell from the trolley as the lady pushed it down the corridor.

Q. What fell from the trolley?

Response: ‘the book’

Information on the right

1. Mary left her diary in the restaurant where she had lunch with Peter.

Q Who did Mary have lunch with?

Response: ‘Peter’.

2. It was four o'clock before the students managed to finish the assignment.

Q. What were the students trying to finish?

Response: ‘the assignment’.

3. The kids regularly went to the park where they loved to play football.

Q. What game did the kids enjoy playing?

Response: ‘football’.

4. The group of friends had planned to spend a weekend shopping in York.

Q. Where did the friends plan to spend the weekend?

Response: ‘York’.

5. They knew that they would only catch him if they called after 8 o'clock.

Q. What time did they need to call after to catch him?

Response: ‘8.00 o-clock’.

6. Jonathon planned a special treat for Sue because it was her birthday.

Q Why did Jonathon plan the treat for Sue?

Response: ‘it was her Birthday’.

7. They regularly went to the pictures after work each week on a Wednesday.

Q. What day did they usually go to the pictures?

Response: ‘Wednesday’.

8. After years of practice Bill became skilled in the sport of ice hockey.

Q. What did Bill become proficient in after practice?

Response: ‘ice hockey’.

9. Shirley looked out of the window where she suddenly saw a large crow.

Q. What did Shirley see out of the window?

Response: ‘large crow’.

10. They had their suspicions, but no one knew for certain who stole the kitty.

Q. What was stolen?

Response: ‘kitty’.
