# Supplementary material for: Eye Movements and Verbal Report in a Single Case of Visual Neglect
Source: PLoS One. 2012 Aug 24;7(8):e43743. doi: 10.1371/journal.pone.0043743 (PMC3427146; doi:10.1371/journal.pone.0043743)
Supplement: Appendix S3 — Includes some movie clips for some trials from the scene inspection and reading studies. (DOCX) [file pone.0043743.s003.docx]

Appendix S3: Movie clips for some trials from the scene inspection and reading studies.

Repin study


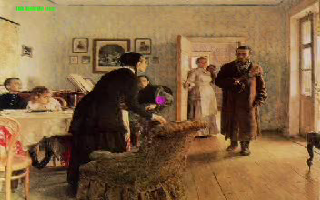


P1_Q7


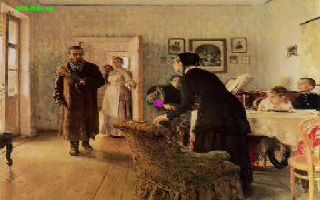


P1_Q9_repeatofQ7


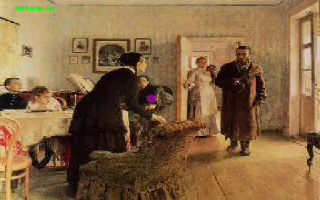


P1_Q8

Reading study


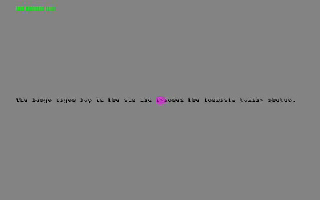

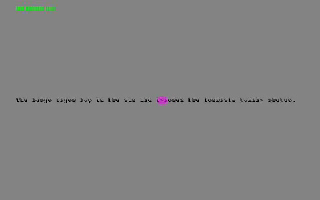


C2

P1
